# Clinical Factors and Cumulative Smoking Burden Associated with Anti-TNF Switching in Radiographic Axial Spondyloarthritis: A Real-World Cohort Study

**DOI:** 10.3390/jcm15218208

**Published:** 2026-10-22

**Authors:** Ahmet Yigitbasi, Baris Yilmazer

**Affiliations:** 1 Department of Internal Medicine, Division of Rheumatology, Trakya University Faculty of Medicine, 22030 Edirne, Türkiye; drbarisyilmazer@hotmail.com; 2 Department of Hematology, Edirne Sultan 1. Murat State Hospital, 22030 Edirne, Türkiye; 3 Department of Rheumatology, İskenderun Gelisim Hospital, 31200 Hatay, Türkiye

**Keywords:** radiographic axial spondyloarthritis, ankylosing spondylitis, tumor necrosis factor inhibitors, smoking, pack-years, treatment persistence, drug survival

## Abstract

**Background/Objectives:** Anti-tumor necrosis factor (anti-TNF) switching remains common in radiographic axial spondyloarthritis (r-axSpA). We aimed to evaluate cumulative smoking burden and clinical factors associated with anti-TNF switching. **Methods:** This retrospective single-center cohort included 212 patients with r-axSpA. Clinical data were collected from medical records. Bivariate comparisons, Kaplan–Meier analysis and multivariable Cox regression assessed switching, with a cause-specific sensitivity analysis for lack-of-response switching. **Results:** Eighty patients (37.7%) switched initial anti-TNF agent, including 70 (87.5%) for lack of response. While the global comparison of smoking status was nonsignificant (*p* = 0.105), exploratory unadjusted comparisons of current smokers versus former and never-smokers combined showed associations with all-cause (*p* = 0.035) and lack-of-response switching (*p* = 0.012). In adjusted Cox analysis, cumulative smoking burden was associated with increased switching hazard (HR 1.025 per pack-year, 95% CI 1.011–1.039; *p* < 0.001), alongside peripheral articular involvement (HR: 2.0, 95% CI 1.216–3.296, *p* = 0.006), higher BASDAI at anti-TNF initiation (HR: 1.02, 95% CI 1.004–1.029, *p* = 0.007) and later calendar year of treatment initiation (HR: 1.24, 95% CI 1.107–1.394, *p* < 0.001). Smoking burden >20 pack-years was associated with a higher adjusted switching hazard compared with never-smokers (HR 3.54, 95% CI 2.081–6.033; *p* < 0.001). Among ever-smokers, Kaplan–Meier comparisons showed shorter drug survival with >20 versus ≤10 and >10–20 pack-years (both *p* < 0.001). **Conclusions:** Cumulative smoking burden was associated with an increased hazard of anti-TNF switching, particularly among patients with >20 pack-years. Quantifying smoking exposure may provide additional information beyond smoking status alone.

## 1. Introduction

Radiographic axial spondyloarthritis (r-axSpA), historically termed ankylosing spondylitis (AS), is a chronic inflammatory disorder that predominantly affects the axial skeleton, particularly the sacroiliac joints and spine, and may progressively lead to structural damage and ankylosis over time [1,2]. Management of r-axSpA relies on an individualized strategy integrating both non-pharmacological and pharmacological interventions. Exercise and patient education remain essential components of care, whereas nonsteroidal anti-inflammatory drugs (NSAIDs) are the first-line pharmacologic treatment, with biologic and targeted synthetic therapies, including tumor necrosis factor inhibitors (TNFis), recommended in patients with persistently active disease despite conventional management [3]. Pivotal randomized controlled trials established the efficacy of anti-TNF agents in active r-axSpA, positioning TNFis as a central therapeutic class in this disease [4,5,6]. Despite their clinical benefits, anti-TNF treatment persistence and discontinuation remain important concerns, prompting investigation of demographic and clinical factors, including age, gender, smoking and disease activity as potential determinants of treatment switching and drug survival [7,8,9,10]. Several validated instruments are used to assess disease activity and functional burden in AS, including the Bath Ankylosing Spondylitis Disease Activity Index (BASDAI), Bath Ankylosing Spondylitis Functional Index (BASFI), Ankylosing Spondylitis Disease Activity Score (ASDAS), and Visual Analog Scale (VAS) [11,12,13]. These measures have also been examined in observational studies of TNFi response and drug survival, highlighting the potential relevance of baseline disease burden to treatment persistence [14,15,16].

Although cumulative smoking exposure has been associated with greater disease activity and functional impairment in r-axSpA in a dose-dependent manner [17], smoking has also been associated with greater cumulative structural damage and radiographic progression [18]. Studies examining TNFi outcomes, however, have largely focused on smoking status rather than quantified cumulative exposure. Patients with the same smoking status may have substantially different exposure histories; pack-years capture both smoking intensity and duration. Large registry studies have demonstrated poorer TNFi response among current smokers, while shorter treatment adherence has also been reported among current and previous smokers [19,20]. However, detailed information on smoking quantity and duration has not been consistently available across these cohorts, limiting assessment of cumulative exposure in relation to TNFi outcomes and treatment persistence.

Switching of TNFi may reflect inadequate response, intolerance or non-adherence; examining both all-cause and lack-of-response switching provides complementary information about treatment persistence. To our knowledge, the association between cumulative smoking exposure quantified by pack-years and anti-TNF treatment switching in r-axSpA has not been specifically investigated. Therefore, the present study aimed to address this gap while also assessing baseline disease activity and other clinical factors in relation to treatment switching.

## 2. Materials and Methods

This retrospective single-center study included 212 patients aged ≥18 years with r-axSpA who had received at least one anti-TNF agent and were followed at the Department of Internal Medicine, Division of Rheumatology, Trakya University Faculty of Medicine. All fulfilled the modified New York criteria [1] and were additionally classified according to the 2009 Assessment of SpondyloArthritis International Society criteria [21]. Patients aged <18 years, those who did not fulfill the diagnostic/classification criteria, and those who had not received anti-TNF treatment were excluded. All eligible patients identified through review of the center’s medical records were included (*n* = 212). Ethical approval was obtained from the Trakya University Scientific Research Ethics Committee (TÜTF-BAEK-2019/17; 4 February 2019), with informed consent waived for retrospective use of anonymized data. Dates of first anti-TNF initiation in the cohort ranged from 2008 through early 2020, while retrospective data abstraction was conducted from February 2019 through early 2020 using historical medical records. Demographic, clinical, laboratory, smoking and treatment data were abstracted. Isolated axial involvement was defined as axial disease without peripheral arthritis, enthesitis, dactylitis, uveitis, IBD or psoriasis. Peripheral articular involvement referred to documented peripheral arthritis. Other non-axial manifestations were recorded separately and could coexist.

Baseline was defined as first anti-TNF initiation. Age, disease activity scores, laboratory measurements and smoking data corresponded to this timepoint. Smoking status, daily cigarette consumption and smoking duration were obtained from detailed patient histories routinely documented before anti-TNF initiation at our center. Cumulative smoking burden was calculated as the number of cigarette packs smoked per day multiplied by the duration of smoking in years. Subsequent assessments were not used to define baseline exposure. Erythrocyte sedimentation rate (ESR, mm/h), C-reactive protein (CRP, mg/L), ASDAS-CRP [13], BASDAI, BASFI (both expressed on 0–100 scales) and pain intensity measured using a 100 mm Visual Analog Scale (VAS; 0 = no pain, 100 = worst imaginable pain) were obtained at first anti-TNF initiation. For calculation of ASDAS-CRP, CRP values < 2 mg/L were assigned a value of 2 mg/L, whereas measured CRP values were retained for laboratory comparisons. The primary outcome was time from initiation of the first anti-TNF agent to the first switch to another anti-TNF agent. Patients remaining on their initial agent at last follow-up were censored. Switching reasons were lack of response, hypersensitivity, infection, or non-compliance; lack of response included primary non-response and secondary loss of response. No discontinuations of the initial anti-TNF agent without subsequent switching were identified. All 80 first switches were to another anti-TNF agent; IL-17 inhibitors were then unavailable at our center. A cause-specific Cox sensitivity analysis of the full cohort (*n* = 212) treated the 70 switches for lack of response as events and censored the remaining 10 at their switching dates.

Statistical analyses were performed using IBM SPSS Statistics version 27.0 (IBM Corp., Armonk, NY, USA). Continuous variables were summarized as mean ± standard deviation or median and categorical variables as numbers (percentages). Distributions were assessed using the Kolmogorov–Smirnov test. Comparisons used Student’s t, Mann–Whitney U, chi-square or Fisher’s exact tests, as appropriate. Exploratory unadjusted Pearson chi-square comparisons assessed current smokers versus former and never smokers combined for all-cause and lack-of-response switching, without adjustment for multiple comparisons. Cumulative smoking burden was determined from smoking histories recorded before treatment initiation, with never-smokers assigned zero pack-years. Multivariable Cox proportional hazards regression assessed time to first anti-TNF switch in the full cohort, adjusting for continuous pack-years, age and BASDAI at first anti-TNF initiation, gender, peripheral articular involvement, initial anti-TNF agent (five categories) and calendar year of initiation. All Cox models used complete covariate data from 212 patients without imputation: the primary model included 80 events and 10 coefficients (8.0 events per coefficient); the model with categorical smoking burden included 80 events and 12 coefficients (6.7 events per coefficient); and the lack-of-response sensitivity model included 70 events and 10 coefficients (7.0 events per coefficient). Patients without switching were censored at their last observation while at risk on the initial agent. Kaplan–Meier analysis assessed first anti-TNF drug survival among ever-smokers grouped as ≤10, >10–20 and >20 pack-years, with pairwise log-rank tests. Median follow-up from first anti-TNF initiation was estimated by reverse Kaplan–Meier analysis, reversing event and censoring indicators. A secondary Cox model in the entire cohort categorized smoking burden as never-smoking (reference), ≤10, >10–20 and >20 pack-years, using the same adjustment covariates. All tests were two-sided; *p* < 0.05 indicated statistical significance.

## 3. Results

Among 212 patients with r-axSpA, the mean overall clinical follow-up from diagnosis was 107.1 ± 55.0 months (median: 108 months), with follow-up data available for all patients. The median observed time from first anti-TNF initiation to first switching or censoring was 60 months (Q1–Q3: 36–96 months), while median follow-up from first anti-TNF initiation, estimated using the reverse Kaplan–Meier method, was 84 months (95% CI 71.3–96.7). Median observation times were 36 months (Q1–Q3: 10.3–60 months) among switchers and 60 months (Q1–Q3: 48–108 months) among non-switchers. According to the initial anti-TNF agent, median observation times were 72 months for infliximab, 48 months for golimumab, 60 months for adalimumab, 72 months for etanercept, and 36 months for certolizumab. Eighty patients (37.7%) underwent anti-TNF switching, whereas 132 (62.3%) continued their initial anti-TNF agent. Twenty-five patients (11.8%) switched more than one anti-TNF agent. Lack of response was the most commonly recorded reason for switching from the initial anti-TNF agent, accounting for 70 of the 80 switches (87.5%). Hypersensitivity (*n* = 4), infection (*n* = 2), and noncompliance (*n* = 4) were less frequent reasons for anti-TNF switching.

Patients who switched and those who continued the initial anti-TNF treatment had similar ages (median (range): 37 (19–61) vs. 39 (22–64); *p* = 0.30) and gender distributions (*p* = 0.29). Comorbid HT was more frequent among patients who switched anti-TNF therapy (*p* = 0.008), whereas DM and CAD were not associated (*p* = 0.85 and *p* = 0.30, respectively). HLA-B27 results were available for only 20/212 patients (9.4%), of whom 19 (95.0%) were positive; owing to the limited availability of these data, HLA-B27 was not included in association analyses. Disease duration < 5 years was less frequent among switchers (11/80 vs. 39/132, *p* = 0.009). At baseline, median ESR (29.0 vs. 28.0 mm/h; *p* = 0.62), CRP (9.5 vs. 9.5 mg/L; *p* = 0.74), and ASDAS (2.5 vs. 2.3; *p* = 0.99) values did not differ significantly between switchers and non-switchers. In contrast, median BASDAI (32 vs. 24; *p* = 0.002) and VAS (50 vs. 40; *p* = 0.018) values were significantly higher among switchers, whereas BASFI was numerically higher without reaching statistical significance (32 vs. 23; *p* = 0.080, Table 1 and Table 2).

**Figure 1:**
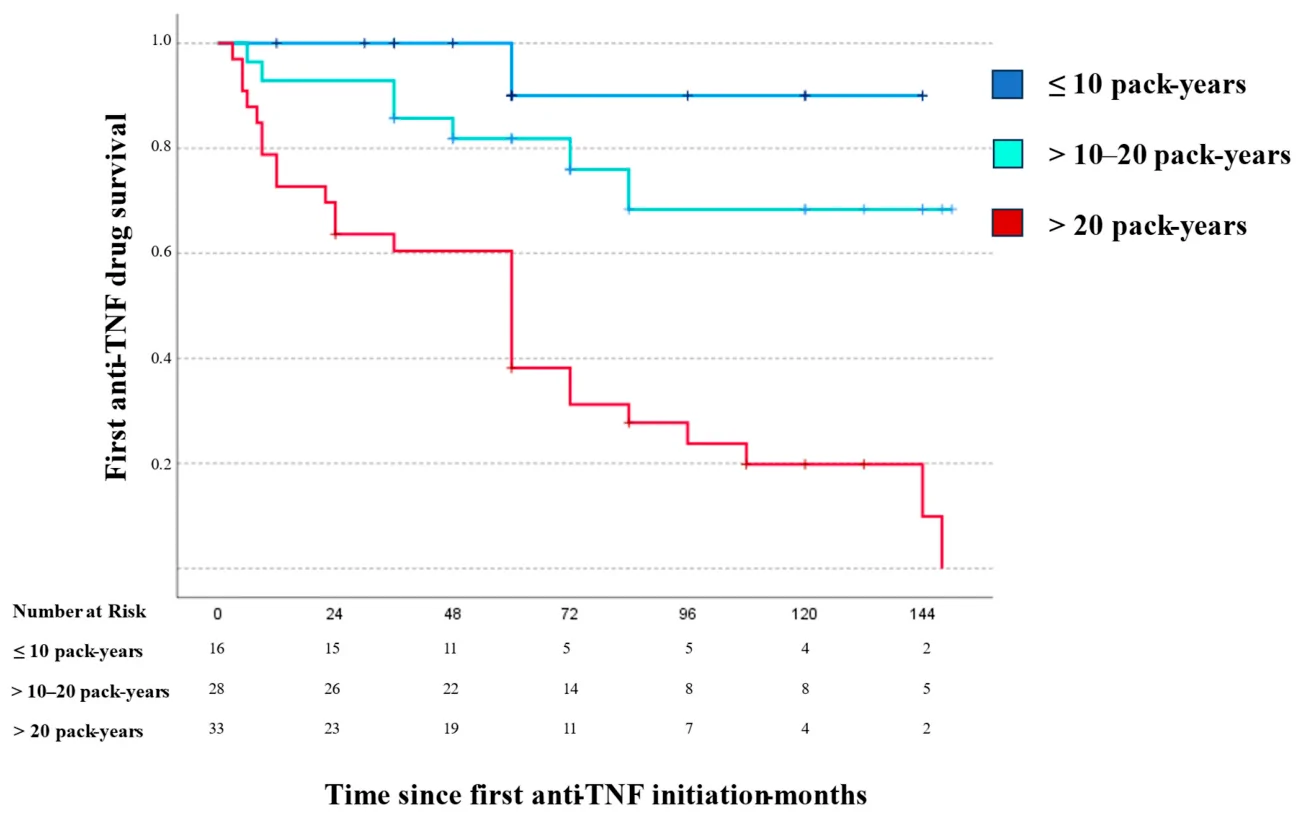
Kaplan–Meier analysis of first anti-TNF drug survival according to cumulative smoking burden among patients with a history of smoking. Footnote: Kaplan–Meier curves show time from first anti-TNF initiation to first anti-TNF switch among ever-smokers (*n* = 77), categorized as ≤10 (*n* = 16), >10–20 (*n* = 28), and >20 pack-years (*n* = 33). Patients remaining on their initial agent at last follow-up were censored; tick marks indicate censored observations. Pairwise log-rank comparisons: ≤10 versus >10–20 pack-years, *p* = 0.193; ≤10 versus >20 pack-years, *p* < 0.001; >10–20 versus >20 pack-years, *p* < 0.001.

The overall distribution of smoking status did not differ significantly between switchers and non-switchers (global *p* = 0.105). Exploratory unadjusted comparisons showed higher proportions of all-cause switching (31/64 (48.4%) vs. 49/148 (33.1%), *p* = 0.035) and lack-of-response switching (29/64 (45.3%) vs. 41/148 (27.7%), *p* = 0.012) in current smokers than in former and never-smokers combined. In the ever-smoker cohort (*n* = 77), switchers had a greater cumulative smoking burden than non-switchers (median: 30 vs. 15 pack-years; *p* < 0.001). First anti-TNF drug survival was shorter in the >20 pack-year group (median: 60 months; 95% CI: 41.9–78.1) than in the ≤10 and >10–20 pack-year groups (pairwise log-rank *p* < 0.001 for both comparisons; Figure 1). Isolated axial involvement was less frequent among switchers (28/80 vs. 79/132, *p* < 0.001), whereas peripheral articular involvement was more frequent (47/80 vs. 42/132, *p* < 0.001). Enthesitis, uveitis, IBD, psoriasis, and concomitant NSAID, sulfasalazine, or MTX use were not associated with switching (all *p* > 0.05; Table 1).

In multivariable Cox proportional hazards regression including all 212 patients, cumulative smoking burden remained associated with an increased hazard of first anti-TNF switching (HR 1.025 per pack-year, 95% CI 1.011–1.039; *p* < 0.001). Peripheral articular involvement (HR 2.002, 95% CI 1.216–3.296; *p* = 0.006), higher BASDAI at first anti-TNF initiation (HR 1.016, 95% CI 1.004–1.029; *p* = 0.007) and later calendar year of treatment initiation (HR 1.242, 95% CI 1.107–1.394; *p* < 0.001) were also significantly associated with switching. The distribution of initial anti-TNF agents differed between switchers and non-switchers in unadjusted analysis (global *p* = 0.037). Age (*p* = 0.149), gender (*p* = 0.062), and initial anti-TNF agent (global Wald *p* = 0.399, Table 3) were not independently associated with switching in the adjusted model. In a secondary multivariable Cox analysis using never-smokers as the reference, smoking burden >20 pack-years was significantly associated with an increased switching hazard (HR 3.544, 95% CI 2.081–6.033; *p* < 0.001). In the cause-specific Cox sensitivity analysis of the full cohort, with 70 switches due to lack of response treated as events, cumulative smoking burden remained associated with an increased switching hazard after adjustment for the same covariates (HR 1.025 per pack-year, 95% CI 1.012–1.038; *p* < 0.001). Peripheral articular involvement (HR 2.157, 95% CI 1.277–3.645; *p* = 0.004), BASDAI at first anti-TNF initiation (HR 1.015, 95% CI 1.003–1.029; *p* = 0.018), and calendar year of treatment initiation (HR 1.207, 95% CI 1.078–1.352; *p* = 0.001) remained associated with response-related switching.

## 4. Discussion

Multiple observational cohorts have evaluated demographic and clinical factors associated with anti-TNF response, switching, and long-term drug survival in r-axSpA [7,8,9,10]. In our cohort, 80 patients (37.7%) switched from their initial anti-TNF agent, comparable to rates reported in previous real-world studies [22]. In unadjusted comparisons, higher BASDAI and VAS scores were associated with switching, whereas CRP, ESR, and ASDAS were not. This discordance may reflect differences between patient-reported symptom burden and measures incorporating acute-phase reactants [23]. Fibromyalgia and central sensitization may contribute by increasing symptom burden without corresponding inflammatory findings and potentially influencing biologic treatment switching [24]. Disease duration <5 years and isolated axial involvement were associated with treatment retention in unadjusted comparisons. Higher BASDAI at first anti-TNF initiation and peripheral articular involvement remained associated with an increased switching hazard in the adjusted full-cohort Cox model, although associations between clinical phenotype and persistence have varied across previous cohorts [7,15,25]. Later calendar year of anti-TNF initiation was also associated with a higher switching hazard. This may reflect unmeasured secular changes in treatment availability and clinical practice over the study period, including broader access to alternative TNF inhibitors.

Smoking has been associated with adverse clinical and structural outcomes in r-axSpA. A dose-dependent association between cumulative smoking exposure and disease burden has been demonstrated, with increasing pack-years associated with greater disease activity and functional impairment [17,26]. Registry studies have also linked smoking status to TNFi outcomes. Glintborg et al. reported shorter treatment adherence among current and previous smokers and poorer TNFi response among current smokers, although exposure was assessed categorically rather than as cumulative burden [19]. More recently, Jones et al. reported a poorer TNFi response in a multinational cohort of 14,885 patients, although their analysis focused primarily on response through 12 months. Pack-year data were available from only three registries, limiting assessment of dose-dependent relationships between cumulative exposure and TNFi outcomes [20]. Evidence linking smoking to TNFi outcomes remains inconsistent: Ciurea et al. reported lower odds of BASDAI50 and ASAS40 response at 1 year in current smokers [27], whereas Zhao et al. found no evidence of an association between baseline smoking status and TNFi discontinuation, although cumulative exposure in pack-years was not assessed [28]. Differences in outcome definitions and smoking assessment may partly explain these findings. Thus, the relationship between cumulative smoking burden and longer-term anti-TNF persistence remains insufficiently characterized.

Although the overall smoking status comparison was nonsignificant, exploratory unadjusted comparisons of current smokers versus former and never smokers combined identified associations with both all-cause and lack of response switching. After multivariable adjustment, increasing pack-year burden remained associated with shorter anti-TNF persistence, with the clearest separation observed among patients exceeding 20 pack-years. Kaplan–Meier analyses among ever-smokers showed shorter treatment persistence predominantly in the >20 pack-year group, while the continuous pack-year association remained significant in the cause-specific analysis restricted to switching due to lack of response, supporting consistency across complementary analyses. Taken together, these converging analyses suggest that the clinically relevant smoking signal may lie less in smoking status itself than in the burden of exposure accrued over time. Quantifying cumulative smoking exposure may therefore provide prognostic information on anti-TNF persistence beyond conventional smoking status, although its incremental predictive value requires formal assessment. This association should be interpreted as a marker of treatment persistence rather than evidence of a direct causal effect on TNFi efficacy, as cumulative exposure may also reflect differences in symptom burden, comorbidity, socioeconomic circumstances, or other unmeasured determinants of treatment persistence.

This study is limited by its retrospective, single-center design and potential residual confounding in treatment switching decisions. Limited baseline BMI data precluded its inclusion in the multivariable analysis. Although detailed smoking histories were routinely documented before anti-TNF initiation, cumulative exposure estimates relied on patient-reported cigarette consumption and smoking duration and were not independently validated. The cohort reflects treatment patterns through early 2020, before the broader availability of newer biologic and targeted synthetic therapies. Nevertheless, it provides real-world data from 212 patients on the association between cumulative smoking burden and anti-TNF persistence. TNF inhibitors remain established treatment options in contemporary r-axSpA management, and treatment switching therefore remains clinically relevant [3,29]. Validation in larger multicenter prospective cohorts is warranted and the observed associations support further evaluation of cumulative smoking burden as a potential marker of anti-TNF persistence.

## Figures and Tables

**Table 1:** Demographic, clinical, and treatment characteristics of patients with radiographic axial spondyloarthritis at first anti-TNF initiation according to subsequent switching status.

Characteristics	Cohort *n* = 212 (%)	Anti-TNF Switched 80 (37.7) *n* (%)	Anti-TNF Continued 132 (62.3) *n* (%)	*p* Values
Male gender, *n* (%)	157 (74)	56 (70)	101 (76.5)	0.29
Age at anti-TNF initiation, years				0.30
median (min–max)	38.5 (19–64)	37 (19–61)	39 (22–64)	
Comorbidity, *n* (%)				
HT	25 (11.8)	16 (20)	9 (6.8)	**0.008**
DM	15 (7)	6 (7.5)	9 (6.8)	0.85
CAD	3 (1.4)	2 (2.5)	1 (0.8)	0.30
Disease duration < 5 years, *n* (%)	50 (23.6)	11 (13.8)	39 (29.5)	**0.009**
Smoking status, *n* (%)				0.105
Never smoker	135 (63.7)	45 (56.3)	90 (68.2)	
Current Smoker	64 (30.2)	31 (38.8)	33 (25)	
Former Smoker	13 (6.1)	4 (5)	9 (6.8)	
Cumulative smoking in ever-smokers,				**<0.001**
median (min–max)	20 (5–80)	30 (10–80)	15 (5–40)	
Clinical manifestation, *n* (%)				
Isolated axial involvement	107 (50.5)	28 (35)	79 (59.8)	**<0.001**
Peripheral articular involvement	89 (42)	47 (58.8)	42 (31.8)	**<0.001**
Enthesitis	32 (15.1)	16 (20)	16 (12.1)	0.12
Uveitis	28 (13.2)	13 (16.3)	15 (11.4)	0.31
IBD	18 (8.5)	6 (7.5)	12 (9.1)	0.69
Psoriasis	2 (0.9)	1 (1.3)	1 (0.8)	0.72
Additional therapy, *n* (%)				
NSAID	200 (94.3)	78 (97.5)	122 (92.4)	0.22
SZP	38 (17.9)	16 (20)	22 (16.7)	0.58
MTX	8 (3.8)	5 (6.3)	3 (2.3)	0.14
Anti-TNF agent, *n* (%)				**0.** **037**
Infliximab	83 (39.2)	35 (43.8)	48 (36.4)	
Golimumab	46 (21.7)	9 (11.3)	37 (28.0)	
Adalimumab	41 (19.3)	16 (20)	25 (18.9)	
Etanercept	35 (16.5)	18 (22.5)	17 (12.9)	
Certolizumab	7 (3.3)	2 (2.5)	5 (3.8)	
Reason for anti-TNF switching, *n* (%)				
Lack of response	-	70 (87.5)	-	-
Hypersensitivity	-	4 (5.0)	-	-
Infection	-	2 (2.5)	-	-
Non-compliance	-	4 (5.0)	-	-

Footnote: Data are presented as median (minimum–maximum) or *n* (%). Baseline refers to first anti-TNF initiation. Percentages use the total number of patients in each column as the denominator. Cumulative smoking burden (pack-years) is summarized only among ever-smokers, comprising current and former smokers (*n* = 77; switched: *n* = 35; continued: *n* = 42). Isolated axial involvement was defined as axial disease without documented peripheral arthritis, enthesitis, dactylitis, uveitis, IBD or psoriasis. Continuous variables were compared using the Mann–Whitney U test and categorical variables using Pearson’s chi-square or Fisher’s exact test, as appropriate. Global *p* values are presented for smoking status and initial anti-TNF agent. Switching reasons refer only to the 80 patients who switched; dashes indicate not applicable. All tests were two-sided; bold *p* values indicate *p* < 0.05. Abbreviations: Anti-TNF: anti-tumor necrosis factor; HT: hypertension; DM: diabetes mellitus; CAD: coronary artery disease; IBD: inflammatory bowel disease; NSAIDs: non-steroidal anti-inflammatory drugs; SZP: sulfasalazine; MTX: methotrexate.

**Table 2:** Comparison of continuous clinical and laboratory variables according to anti-TNF switching.

Predictor Median (Min–Max)	Anti-TNF Status				
	Switched *n* = 80	Continued *n* = 132	U	Z	*p*
ESR at baseline, mm/h	29 (2–100)	28 (2–132)	5066	−0.494	0.62
CRP at baseline, mg/L	9.5 (1.7–60)	9.5 (1.5–60)	5139	−0.327	0.74
BASDAI at baseline	32 (0–86)	24 (0–83)	3970	−3.033	**0.002**
VAS at baseline	50.0 (0–100)	40.0 (0–100)	4261	−2.367	**0.018**
ASDAS-CRP at baseline	2.5 (0.6–5.4)	2.3 (0.6–4.7)	5279	−0.002	0.99
BASFI at baseline	32.0 (0–88)	23 (0–96)	4525	−1.744	0.08
Smoking burden, pack-years	30 (10–80)	15 (5–40)	214	−5.39	**<0.001**

Footnote: Continuous variables are presented as median (minimum–maximum). Baseline values correspond to values at first anti-TNF initiation. BASDAI, BASFI and VAS scores were expressed on 0–100 scales. Comparisons between patients who switched anti-TNF therapy and those who continued treatment were performed using the Mann–Whitney U test. Smoking burden was analyzed only among ever-smokers (*n* = 77; switched: *n* = 35, continued: *n* = 42). U statistics, Z values, and two-sided *p* values are presented. Bold *p* values indicate statistically significant differences (*p* < 0.05). Abbreviations: Anti-TNF: anti-tumor necrosis factor; ESR: erythrocyte sedimentation rate; CRP: C-reactive protein; BASDAI: Bath Ankylosing Spondylitis Disease Activity Index; VAS: Visual Analog Scale; ASDAS: Ankylosing Spondylitis Disease Activity Score; BASFI: Bath Ankylosing Spondylitis Functional Index.

**Table 3:** Multivariable Cox proportional hazards regression analysis of factors associated with time to first anti-TNF switching in patients with radiographic axial spondyloarthritis.

Characteristics *n* = 212	Entire Cohort *n* = 212		
	*p* Value	HR	95% CI
Age at Anti-TNF initiation, years	0.149	0.982	0.957–1.007
Male gender	0.062	0.613	0.366–1.025
BASDAI at anti-TNF initiation	**0.007**	1.016	1.004–1.029
Peripheral articular involvement	**0.006**	2.002	1.216–3.296
Calendar year of first anti-TNF initiation	**<0.001**	1.242	1.107–1.394
Initial anti-TNF agent	0.399	-	-
Smoking burden, pack-years	**<0.001**	1.025	1.011–1.039

Footnote: Multivariable Cox proportional hazards regression included the entire cohort (*n* = 212), with first anti-TNF switching defined as the event (*n* = 80). Patients without switching (*n* = 132) were censored at their last documented observation on the initial anti-TNF agent. Variables entered simultaneously were age at first anti-TNF initiation, gender, BASDAI at first anti-TNF initiation, peripheral articular involvement, calendar year of first anti-TNF initiation, initial anti-TNF agent, and cumulative smoking burden modeled continuously in pack-years. Initial anti-TNF agent was included as a five-category covariate, with its overall association assessed using a four-degree-of-freedom Wald test. Never-smokers were assigned zero pack-years and retained in the analysis. Hazard ratios (HRs) with 95% confidence intervals (CIs) are presented. Bold *p* values indicate statistically significant differences (*p* < 0.05). Dashes indicate that a single HR and CI are not applicable to the overall test of the five-category initial anti-TNF variable. Abbreviations: Anti-TNF: anti-tumor necrosis factor; BASDAI: Bath Ankylosing Spondylitis Disease Activity Index; HR: hazard ratio; CI: confidence interval.

## Data Availability

The data presented in this study are available on request from the corresponding author due to privacy and ethical restrictions and subject to institutional approval.

## References

[1] van der Linden S., Valkenburg H.A., Cats A. (1984). Evaluation of diagnostic criteria for ankylosing spondylitis: A proposal for modification of the New York criteria. Arthritis Rheum..

[2] Van Mechelen M., Gulino G.R., de Vlam K., Lories R.J. (2018). Bone disease in axial spondyloarthritis. Calcif. Tissue Int..

[3] Ramiro S., Nikiphorou E., Sepriano A., Ortolan A., Webers C., Baraliakos X., Landewé R.B.M., Van den Bosch F.E., Boteva B., Bremander A. (2023). ASAS–EULAR recommendations for the management of axial spondyloarthritis: 2022 update. Ann. Rheum. Dis..

[4] van der Heijde D., Dijkmans B., Geusens P., Sieper J., DeWoody K., Williamson P., Braun J. (2005). Efficacy and safety of infliximab in patients with ankylosing spondylitis: Results of a randomized, placebo-controlled trial (ASSERT). Arthritis Rheum..

[5] van der Heijde D., Kivitz A., Schiff M.H., Sieper J., Dijkmans B.A., Braun J., Dougados M., Reveille J.D., Wong R.L., Kupper H. (2006). Efficacy and safety of adalimumab in patients with ankylosing spondylitis: Results of a multicentre, randomized, double-blind, placebo-controlled trial. Arthritis Rheum..

[6] Davis J.C., van der Heijde D., Braun J., Dougados M., Cush J., Clegg D.O., Kivitz A., Fleischmann R., Inman R., Tsuji W. (2003). Recombinant human tumour necrosis factor receptor (etanercept) for treating ankylosing spondylitis: A randomized, controlled trial. Arthritis Rheum..

[7] Kristensen L.E., Karlsson J.A., Englund M., Petersson I.F., Saxne T., Geborek P. (2010). Presence of peripheral arthritis and male sex predicting continuation of anti-tumour necrosis factor therapy in ankylosing spondylitis: An observational prospective cohort study from the South Swedish Arthritis Treatment Group Register. Arthritis Care Res..

[8] Lee J.W., Kang J.H., Yim Y.R., Kim J.E., Wen L., Lee K.E., Park D.-J., Kim T.-J., Park Y.-W., Lee S.-S. (2015). Predictors of switching anti-tumour necrosis factor therapy in patients with ankylosing spondylitis. PLoS ONE.

[9] Glintborg B., Østergaard M., Krogh N.S., Dreyer L., Kristensen H.L., Hetland M.L. (2010). Predictors of treatment response and drug continuation in 842 patients with ankylosing spondylitis treated with anti-tumour necrosis factor: Results from 8 years’ surveillance in the Danish nationwide DANBIO registry. Ann. Rheum. Dis..

[10] Yahya F., Gaffney K., Hamilton L., Lonsdale E., Leeder J., Brooksby A., Cavill C., Berry-Jenkins J., Boyle C., Bond D. (2018). Tumour necrosis factor inhibitor survival and predictors of response in axial spondyloarthritis: Findings from a United Kingdom cohort. Rheumatology.

[11] Garrett S., Jenkinson T., Kennedy L.G., Whitelock H., Gaisford P., Calin A. (1994). A new approach to defining disease status in ankylosing spondylitis: The Bath Ankylosing Spondylitis Disease Activity Index. J. Rheumatol..

[12] Calin A., Garrett S., Whitelock H., Kennedy L.G., O’Hea J., Mallorie P. (1994). A new approach to defining functional ability in ankylosing spondylitis: The development of the Bath Ankylosing Spondylitis Functional Index. J. Rheumatol..

[13] Lukas C., Landewé R., Sieper J., Dougados M., Davis J., Braun J., van der Linden S., van der Heijde D. (2009). Development of an ASAS-endorsed disease activity score (ASDAS) in patients with ankylosing spondylitis. Ann. Rheum. Dis..

[14] Lopalco G., Venerito V., Cantarini L., Emmi G., Salaffi F., Di Carlo M., Tafuri S., Gentileschi S., Di Scala G., Nivuori M. (2019). Different drug survival of first-line tumour necrosis factor inhibitors in radiographic and non-radiographic axial spondyloarthritis: A multicentre retrospective survey. Clin. Exp. Rheumatol..

[15] Arends S., Brouwer E., van der Veer E., Groen H., Leijsma M.K., Houtman P.M., Jansen T.L.T.A., Kallenberg C.G., Spoorenberg A. (2011). Baseline predictors of response and discontinuation of tumour necrosis factor-alpha blocking therapy in ankylosing spondylitis: A prospective longitudinal observational cohort study. Arthritis Res. Ther..

[16] Lorenzin M., Ortolan A., Frallonardo P., Oliviero F., Punzi L., Ramonda R. (2015). Predictors of response and drug survival in patients with ankylosing spondylitis treated with infliximab. BMC Musculoskelet. Disord..

[17] Zhao S., Challoner B., Khattak M., Moots R.J., Goodson N.J. (2017). Increasing smoking intensity is associated with increased disease activity in axial spondyloarthritis. Rheumatol. Int..

[18] Solmaz D., Gercik Ö., Gücenmez S., Akar S., Keskin-Arslan E., Kaplan Y.C., Ecemiş S. (2019). The role of smoking in the development and progression of structural damage in axial SpA patients: A systematic review and meta-analysis. Eur. J. Rheumatol..

[19] Glintborg B., Højgaard P., Lund Hetland M., Krogh N.S., Kollerup G., Jensen J., Chrysidis S., Hansen I.M.J., Holland-Fischer M., Hansen T.H. (2016). Impact of tobacco smoking on response to tumour necrosis factor-alpha inhibitor treatment in patients with ankylosing spondylitis: Results from the Danish nationwide DANBIO registry. Rheumatology.

[20] Jones G.T., Rotariu O., MacDonald R., Michelsen B., Glintborg B., van der Horst-Bruinsma I.E., Gudbjornsson B., Geirsson A.J., Relas H., Isomäki P. (2025). The relationship between lifestyle factors and outcome of treatment with TNFα inhibitors in axial spondyloarthritis—Results from 14 European countries. BMC Rheumatol..

[21] Rudwaleit M., van der Heijde D., Landewé R., Listing J., Akkoc N., Brandt J., Braun J., Chou C.T., Collantes-Estevez E., Dougados M. (2009). The development of Assessment of SpondyloArthritis international Society classification criteria for axial spondyloarthritis, part II: Validation and final selection. Ann. Rheum. Dis..

[22] Glintborg B., Østergaard M., Krogh N.S., Tarp U., Manilo N., Loft A.G.R., Hansen A., Schlemmer A., Fana V., Lindegaard H.M. (2013). Clinical response, drug survival, and predictors thereof in 432 patients with ankylosing spondylitis after switching tumour necrosis factor-α inhibitor therapy: Results from the Danish nationwide DANBIO registry. Ann. Rheum. Dis..

[23] Michielsens C., Bolhuis T.E., van Gaalen F.A., van den Hoogen F.H.J., Verhoef L.M., den Broeder N., Broeder A.D. (2024). Construct validity of Bath Ankylosing Spondylitis Disease Activity Index and Ankylosing Spondylitis Disease Activity Score treatment-target cut-offs in a BASDAI treat-to-target axial spondyloarthritis cohort: A cross-sectional study. Scand. J. Rheumatol..

[24] Eltoum H.A., Hashim N.T.O., Mohammed S.A., Hassanelbasri D.G., Ahmed A., Alamin M.A. (2026). Fibromyalgia and Central Sensitization in Rheumatoid Arthritis, Psoriatic Arthritis, and Spondyloarthritis: A Systematic Review. Cureus.

[25] Flouri I.D., Markatseli T.E., Boki K.A., Papadopoulos I., Skopouli F.N., Voulgari P.V., Settas L., Zisopoulos D., Iliopoulos A., Geborek P. (2018). Comparative analysis and predictors of 10-year tumour necrosis factor inhibitor drug survival in patients with spondyloarthritis: First-year response predicts long-term drug persistence. J. Rheumatol..

[26] Zhang H., Wan W., Liu J., Dai S.M., Zou Y.H., Qian Q.X., Ding Y., Xu X., Ji H., He H. (2018). Smoking quantity determines disease activity and function in Chinese patients with ankylosing spondylitis. Clin. Rheumatol..

[27] Ciurea A., Scherer A., Weber U., Exer P., Bernhard J., Tamborrini G., Riek M., Müller R.B., Weiss B., Nissen M.J. (2016). Impaired response to treatment with tumour necrosis factor α inhibitors in smokers with axial spondyloarthritis. Ann. Rheum. Dis..

[28] Zhao S.S., Yoshida K., Jones G.T., Hughes D.M., Duffield S.J., Tedeschi S.K., Lyu H., Moots R.J., Solomon D.H., Goodson N.J. (2019). Smoking status and cause-specific discontinuation of tumour necrosis factor inhibitors in axial spondyloarthritis. Arthritis Res. Ther..

[29] Zhao S.S., Harrison S.R., Thompson B., Yates M., Eddison J., Chan A., Clarke N., Corp N., Davis C., Felix L. (2025). The 2025 British Society for Rheumatology guideline for the treatment of axial spondyloarthritis with biologic and targeted synthetic DMARDs. Rheumatology.

